# Regulation of Plant Tannin Synthesis in Crop Species

**DOI:** 10.3389/fgene.2022.870976

**Published:** 2022-05-02

**Authors:** José Mora, Delphine M. Pott, Sonia Osorio, José G. Vallarino

**Affiliations:** Instituto de Hortofruticultura Subtropical y Mediterránea “La Mayora”—Consejo Superior de Investigaciones Científicas-Universidad de Málaga- (IHSM-CSIC-UMA), Málaga, Spain

**Keywords:** antioxidants, flavonoids, proanthocyanidins, fruits, biosynthesis, ellagitannins (ETs)

## Abstract

Plant tannins belong to the antioxidant compound family, which includes chemicals responsible for protecting biological structures from the harmful effects of oxidative stress. A wide range of plants and crops are rich in antioxidant compounds, offering resistance to biotic, mainly against pathogens and herbivores, and abiotic stresses, such as light and wound stresses. These compounds are also related to human health benefits, offering protective effects against cardiovascular and neurodegenerative diseases in addition to providing anti-tumor, anti-inflammatory, and anti-bacterial characteristics. Most of these compounds are structurally and biosynthetically related, being synthesized through the shikimate-phenylpropanoid pathways, offering several classes of plant antioxidants: flavonoids, anthocyanins, and tannins. Tannins are divided into two major classes: condensed tannins or proanthocyanidins and hydrolysable tannins. Hydrolysable tannin synthesis branches directly from the shikimate pathway, while condensed tannins are derived from the flavonoid pathway, one of the branches of the phenylpropanoid pathway. Both types of tannins have been proposed as important molecules for taste perception of many fruits and beverages, especially wine, besides their well-known roles in plant defense and human health. Regulation at the gene level, biosynthesis and degradation have been extensively studied in condensed tannins in crops like grapevine (*Vitis vinifera*), persimmon (*Diospyros kaki*) and several berry species due to their high tannin content and their importance in the food and beverage industry. On the other hand, much less information is available regarding hydrolysable tannins, although some key aspects of their biosynthesis and regulation have been recently discovered. Here, we review recent findings about tannin metabolism, information that could be of high importance for crop breeding programs to obtain varieties with enhanced nutritional characteristics.

## Introduction

Antioxidant compounds are chemical species whose function is to inhibit or delay the process of oxidation (Mittler, 2017). Oxidation is the chemical process of losing electrons, but biologically speaking, it is the production of free radical (reactive) species, which can attack electron-rich molecules such as lipids, proteins, or even nucleic acids, damaging cells and tissues, and thereby causing the alteration of homeostasis (Engwa, 2018). To fight against these reactive species, plants have a myriad of chemical compounds known as bio-active molecules whose activities are based on their antioxidant properties. These compounds have the ability to protect biological structures by acting as scavengers of free radicals such as reactive oxygen species (ROS) and reactive nitrogen species (RNS) (Karadag et al., 2009; Wang et al., 2009).

One of the main chemical classes of plant antioxidants are polyphenol (phenolic) compounds. These metabolites are mainly composed of aromatic rings with hydroxyl groups capable of reacting with peroxide radicals to block degradation reactions (Hyun et al., 2010). They are major and ubiquitous secondary metabolites derived from phenylalanine and shikimate pathways (Quideau et al., 2011). Polyphenols range from simple molecules such as phenolic acids to more complex structures that are highly polymerized metabolites, including tannins, flavonoids, and lignans or stilbenes (Pott et al., 2019).

The study of plant polyphenols started thanks to the work of four scientists: Theodore White, E. C. Bate-Smith, Tony Swain, and Edwin Haslam. According to their definition of plant polyphenols, only monosubstituted phenols or compounds having di- and/or tri-hydroxyphenyl moieties can fit the definition of “true plant polyphenol” (Haslam and Cai, 1994). Given that, only proanthocyanidins and hydrolysable tannins, which exclude lignin polymers among others, are considered true plant polyphenols (Haslam and Cai, 1994). According to Quideau et al. (2011), a polyphenol is a plant secondary metabolite derived from the phenylpropanoid-shikimate and polyketide pathways that has more than one phenolic unit and is deprived of any nitrogen-based functional group. Nowadays, it is widely accepted that a polyphenol is a naturally occurring compound composed of several aromatic rings substituted with hydroxyl groups. This definition comprises four principal groups of polyphenols: flavonoids, phenolic acids, lignans, and stilbenes (Zhou et al., 2016).

An important class of polyphenols is tannins, which have been used for ages in the treatment of animal skins for leather manufacturing and to prevent putrefaction. The reason is the ability of tannins to interact with proteins, stabilizing them and turning the skin tanned in order to be transformed into leather (Baxter et al., 1997; Falcão and Araújo, 2018). The term “tannin” includes three families of compounds: condensed tannins (proanthocyanidins), hydrolysable tannins (gallotannins and ellagitannins) and phlorotannins (only found in marine red-brown algae). According to Zhou et al. (2016) classification, condensed tannins belong to flavonoid-related compounds, while ellagitannins belong to phenolic acid-related compounds. Tannins can be found naturally in plants and their main function is to act as a natural barrier against pathogens or herbivores (Haslam and Cai, 1994). The consumption of fruits enriched in polyphenols, such as berries, pomegranates (*Punica granatum*), persimmons (*Diospyros kaki*) and nuts, is related to health benefits (Basu et al., 2014; Cory et al., 2018). Of course, tannins, as polyphenols, play a role in crop health-promoting benefits.

The aim of this review is to summarize the genetic factors underlying the regulation of hydrolysable and condensed tannin biosynthesis, respectively. For this purpose, we focus on economically-relevant species, such as kaki, grape (*Vitis vinifera*) or strawberry (*Fragaria* x *ananassa*). These crops are known for their high tannin content and metabolites that significantly contribute to their antioxidant and organoleptic properties. In this sense, we also discuss how the recent findings about the genetic regulation of tannin synthesis may pave the way to breeding new varieties with enhanced nutritional value.

## Biosynthetic Pathways Leading to the Formation of Polyphenol Compounds

**FIGURE 1:**
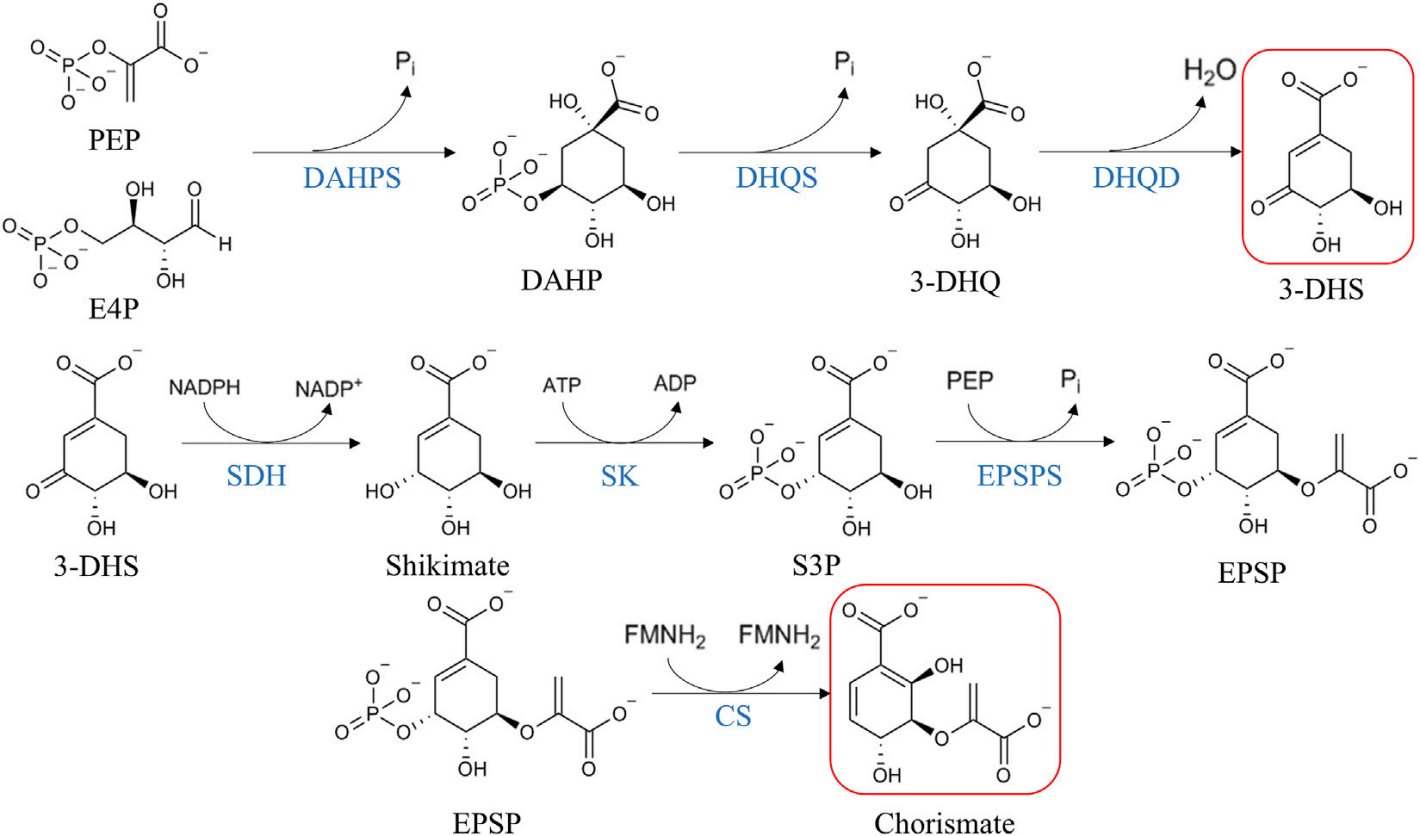
Schematic of shikimate pathway reactions. 3-dehydroshikimate and chorismate are highlighted in red as the precursors of hydrolysable tannins and proanthocyanidins, respectively. Abbreviations: phosphoenolpyruvate (PEP), erythrose 4-phosphate (E4P), inorganic phosphate (P_i_), 3-deoxy-D-arabino-heptulosonate-7-phosphate synthase (DAHPS), 3-deoxy-D-arabino-heptulosonate-7-phosphate (DAHP), dehydroquinate synthase (DHQS), 3-dehydroquinate (3-DHQ), 3-dehydroquinate dehydratase (DHQD), 3-dehydroshikimate (3-DHS), shikimate dehydrogenase (SDH), shikimate kinase (SK), shikimate-3-phosphate (S3P), 5-enolpyruvylshikimate 3-phosphate synthase (EPSPS), 5-enolpyruvylshikimate-3-phosphate (EPSP), and chorismate synthase (CS). Adapted from Vogt, 2010; Akagi et al., 2011; Mir et al., 2015; Stefanachi et al., 2018.

Many aromatic compounds, and by extension, many antioxidant compounds, are produced by *de novo* synthesis via the shikimate pathway in plants (Bentley and Haslam, 1990). They are produced in plastids, where this pathway is functional. Chorismate is synthetized through seven enzymatic reactions starting from phospho*enol*pyruvate and erythrose-4-phosphate (Figure 1), and is finally transformed into the three aromatic amino acids, L-tyrosine, L-tryptophan, and L-phenylalanine (Mir et al., 2015). L-Phenylalanine is the precursor of many antioxidant compounds like flavonoids, isoflavonoids, phenylpropenes, aurones, coumarins, and phenylpropanoid esters (Stefanachi et al., 2018). Another interesting intermediate of the shikimate pathway is the 3-dehydroshikimate, which starts the biosynthesis of hydrolysable tannins (Vogt, 2010; Bontpart et al., 2016).

The first step of the shikimate pathway is an aldol condensation catalyzed by 3-deoxy-D-arabino-heptulosonate-7-phosphate (DAHP) synthase, taking phospho*enol*pyruvate (PEP) and erythrose-4-phosphate as substrates, resulting in the seven-carbon keto acid DAHP and inorganic phosphate (Mir et al., 2015). In plants, DAHP synthase is regulated transcriptionally by certain environmental stimuli like mechanical wounding or biotic stresses like insects’ attacks. Under these stress conditions, there is an accumulation of mRNA encoding DAHP synthase (Dyer et al., 1989), strengthening the idea of the role of aromatic compounds in plant defense. In addition, DAHP synthase is controlled by feedback inhibition loops, limiting the carbon flux from primary metabolism to polyphenol synthesis (Pott et al., 2019).

The next step is the transformation of DAHP into 3-dehydoquinate, catalyzed by dehydroquinate synthase (DHQS). This enzyme is a metalloenzyme nicotinamide adenine di-nucleotide (NAD^+^)-dependent that acts as an enzymatic complex in the five-step transformation of DAHP. In this multistep mechanism, several reactions occur spontaneously, but only a single site catalyzes five sequential reactions. NAD^+^ is required for the oxidation of C5 of DAHP and the NADH produced is then used in the reduction of the C5 carbonyl intermediate in DAHP (not shown in Figure 1) (Mir et al., 2015).

Then, 3-dehydroquinate is converted to 3-dehydroshikimate due to the presence of 3-dehydroquinate dehydratase (DHQase). This enzyme exists in two types: type I and type II, but with no sequential or structural homology and with no similar reaction mechanisms between them. Type I DHQase (susceptible to thermal denaturation) is found in plants, fungi and some bacteria (Chaudhuri et al., 1986) and this type only participates in biosynthetic reactions, whereas type II DHQase (resistant to thermal denaturation) can participate either in biosynthetic and catabolic reactions (White et al., 1990). Type I enzymes catalyze *cis*-dehydration of 3-dehydroquinate, involving the formation of a Schiff-base intermediate (Shneier et al., 1991), where the conserved amino acids lysine and histidine of the active site play a critical catalytic dyad in generating the carbanion intermediate essential for the course of the reaction (Mir et al., 2015).

The fourth enzyme of the shikimate pathway is the shikimate dehydrogenase (SDH) that acts in the reduction of 3-dehydroshikimate to shikimate by means of the transformation of NADPH to NADP^+^. This enzyme exists in a bifunctional complex in plants (Chaudhuri and Coggins, 1985; Coggins et al., 1987). The catalysis consists of an acid-base mechanism in which a lysine and an aspartate residue form a catalytic dyad. The lysine function is to deprotonate the 3-hydroxyl group of shikimate, its amino group acting as a base (Mir et al., 2015).

Shikimate kinase (SK) is the enzyme responsible for the fifth step of the shikimate pathway. This step is the phosphorylation of shikimate to shikimate-3-phosphate by converting ATP to ADP. The SK is a simple protein (26 kDa in *Arabidopsis thaliana*) that is composed of three domains: the core domain, the lid domain, and the shikimate binding domain. Within the shikimate binding domain, there are several highly conserved amino acids involved in the interaction with the substrate (Mir et al., 2015).

The penultimate step of the pathway is catalyzed by 5-enolpyruvylshikimate 3-phosphate (EPSPS). This is a monomer protein responsible for the reversible transfer of an *enol*pyruvoyl moiety from phospho*enol*pyruvate (PEP) to shikimate-3-phosphate, giving EPSP and phosphate as reversible products. It is remarkable that in this reaction, which proceeds with the cleavage of the C-O bond of PEP, not with the cleavage of the high energy P-O bond (Mir et al., 2015).

Finally, the seventh and last reaction of the shikimate pathway is the one catalyzed by chorismate synthase. In this reaction, EPSPS is converted into chorismate. Although during the course of the reaction there is no redox change (Mir et al., 2015), this enzyme requires the presence of FMNH_2_. The presence of flavin mononucleotide may have two explanations: 1) the flavin has merely a structural role or 2) flavin restores the reduced form of sulfhydryl group structurally or catalytically important for the enzyme (Hasan and Nester, 1978).

Regulation of this pathway occurs mainly at gene expression. The cDNA of higher plants results in proteins with an amino-terminal signal sequence for plastid import. The expression of some plant genes encoding enzymes that participate in the shikimate pathway is regulated during development (Benfey and Chua, 1989; Maeda et al., 2010) and by certain stimuli like wounding (Dyer et al., 1989; Keith et al., 1991), elicitors or pathogens (Keith et al., 1991; Görlach et al., 1995). At a protein level, in higher plants the enzymes possess an amino-terminal sequence required for plastid import, suggesting that the synthesis of chorismate only takes place inside the plastids (Herrmann and Weaver, 1999).

## Tannins and Their Properties: Antioxidants and Relationships with Human Health and Organoleptic Characteristics

The antioxidant properties of tannins have been thoroughly examined from natural sources or from food grade preparations. For example, Shin et al. (2014) studied the antioxidant activities of both hydrolysable and condensed tannins in three cultivars of persimmon. The radical scavenging activity (RSA) is often used to determine the antioxidant capacities and particularly, the 2,2′-azinobis-3-ethylbenzthiazolin-6-sulfonic acid (ABTS) RSA assay allows to measure the antioxidant activities of both hydrophilic and hydrophobic antioxidant chemicals (Floegel et al., 2011). Interestingly, 1,000 μg/ml of soluble tannins from three persimmon cultivars showed higher ABTS RSA than 100 μg/ml of L-ascorbic acid (Shin et al., 2014). The 2,2-diphenyl-1-picrylhydrazyl (DPPH) RSA assay (Blois, 1958) was also conducted in the same study to provide evidence that the DPPH RSA of unripe persimmons was significantly higher than those of ripe persimmons, mirroring the trend of tannin accumulation, which decreases during fruit ripening. Additionally, Ricci et al. (2016) studied the antioxidant activity of commercial food grade tannins (extracted from grape and *Quercus* tissues) in wine samples. Samples with higher tannin content (measured as (+)-catechin equivalents) showed, in most cases, the highest values of DPPH radical scavenging activity. The reducing power of the samples, estimated with the ferric-reducing antioxidant power (FRAP) assay (Benzie and Strain, 1999), ranged between 0.513 and 0.662 mM of FeSO_4_∙7H_2_O, providing effective reducing properties in some cases. Finally, and at least for wine, it seems that based on ABTS, FRAP, and DPPH assays, hydrolysable tannins show higher antioxidant activity than condensed tannins (Pascual et al., 2017; Vignault et al., 2018).

The use of tannins in food and beverages provides certain advantages, like protection from oxidation processes. In the industry, tannins are used as flavoring agents, and, for example, commercial tannins are added for the clarification of grape musts and wines (Ricci et al., 2016). This helps improve the color stability and avoid oxidation (Versari et al., 2013), offering a natural alternative to synthetic antioxidants. The oxidation of certain compounds plays a crucial role in the flavor of some beverages like beer, especially during aging processes (Vanderhaegen et al., 2006). To further confirm the effect of tannins on beverages, De Francesco et al. (2020) added various tannin-rich extracts to beer. This addition led to an improvement in turbidity, color formation, foam quality, citrus and spicy notes, and an increase in the body of the beer.

As previously mentioned, the antioxidant capacities of tannins are of high importance for human health, providing neuroprotective, cardioprotective, and even antitumoral activities. In a recent review, Maugeri et al. (2022) summarized the *in vitro* and *in vivo* effects of tannins in several experimental models and provided evidence of anti-bacterial and anti-inflammatory effects. It can be seen that the anti-inflammatory effects of tannins are related to palliating oxidative stress, reducing ROS and NOS, avoiding DNA damage, and taking part in some signaling routes (Maugeri et al., 2022). In addition, the health benefits of tannins have been thoroughly reviewed elsewhere (Basu et al., 2014; Cory et al., 2018; Lu et al., 2021).

Tannins are present in foods and beverages, providing them with the perception of astringency, which is felt as a sensation of dryness in the mouth and tongue. This event is thought to be due to the precipitation of oral proteins and mucopolysaccharides when they interact with tannins (Baxter et al., 1997). The mechanism of astringency perception has been widely discussed and some hypotheses have been proposed: 1) interaction between tannins and epithelial receptors (Ma et al., 2014), 2) physical movement of mouth musculature (Pires et al., 2020) or 3) increasing of the oral friction caused by protein-tannins aggregates (Rossetti et al., 2009). As tannins are widely distributed among plants, astringency is also present in many food-sources, such as rice (Shao et al., 2018), tea (Xu et al., 2018), grape (Sun et al., 2020), cocoa (Misnawi et al., 2005), and also in some cereals like sorghum and millet (Ahmad et al., 2018). Nevertheless, not all plant-sources have the same quantity of tannins. In the case of proanthocyanidins, the amount of (+)-catechin in some sorghum and millet varieties ranges from 172 to 179 mg/100 g for sorghum and from 172 to 174 mg/100 g (Ahmad et al., 2018), respectively. On the other hand, 11–54 mg/g of (+)-catechin were found in eight different cultivars of persimmon (Akagi et al., 2010b). Concerning ellagitannins, the content in strawberry fruits was around 2–18 mg/g (Karlińska et al., 2021).

Interestingly, astringency is often accompanied by bitterness, which is a source of confusion between both taste properties (Arnold et al., 1980; Green, 1993; Carpenter et al., 2019). However, the perception of astringency seems to be a secondary attribute when both astringency and bitterness are simultaneously perceived in the same product (Arnold et al., 1980; Green, 1993). Interestingly, the astringency/bitterness properties of tannins depend on their degree of polymerization. Molecules with low molecular weight tend to be bitterer, whereas tannins with a high degree of polymerization appear to be more astringent (Robichaud and Noble, 1990; Peleg et al., 1999; Hufnagel and Hofmann, 2008). There is a real challenge in enhancing the tannin content of foods and beverages because of their modulation in organoleptic characteristics, which can lead to non-pleasing foodstuffs. It is needed to better understand the food matrix effects on the perception of astringency and bitterness and to discover new ways to modulate tannins’ contributions to taste perception while maintaining or even increasing these health-promoting compounds.

Some research has already been carried out to solve this particular conflict. For example, the addition of some proteins leads to the removal of highly reactive tannins and the modification of astringency and bitterness (Siebert et al., 1996; Siebert, 1999). Proteins like β-casein, β-lactoglobullin, Ca-caseinate, Na-caseinate, and different types of gelatins have been assayed for their potential bitter-taste-masking (Bohin et al., 2012). For example, the addition of gelatin to red wines causes a decrease in astringency due to the specific removal of high molecular weight galloylated proanthocyanidins (Sarni-Manchado et al., 1999). The contribution of polysaccharides to astringency is also important. Soluble polysaccharides, derived, for example, from pectins during the ripening process, can compete with salivary proteins for polyphenolic substrates like tannins, which lead to a modification in astringency perception (Ozawa et al., 1987).

## Proanthocyanidins or Condensed Tannins

**FIGURE 2:**
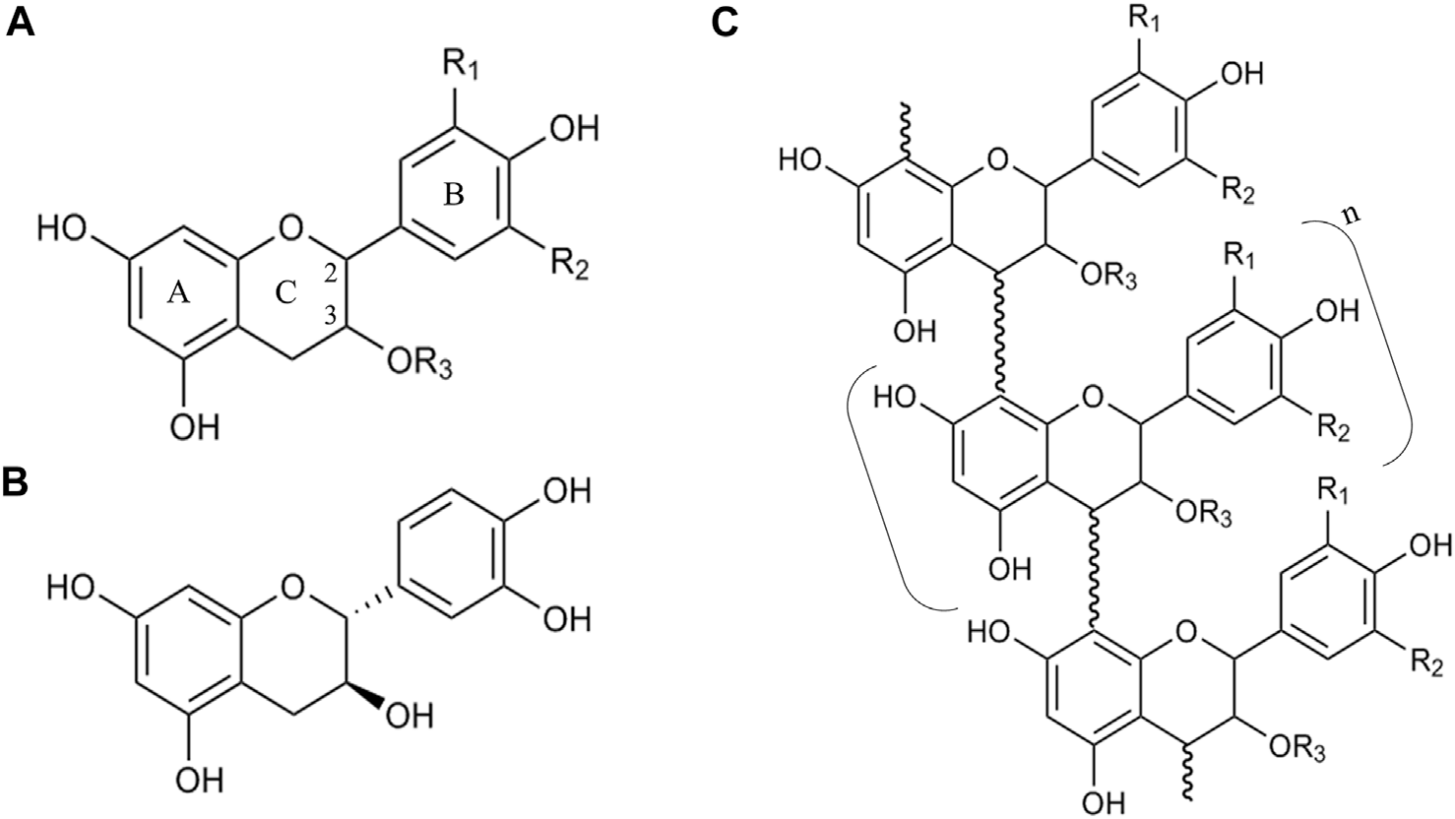
Schematic of the structure of catechins and proanthocyanidins. **(A)** General structure of a catechin, where **(A–C)** indicate the distinct rings of the carbon backbone C6-C3-C6, numbers 2 and 3 indicate the chiral carbons (stereochemistry not shown), and R_1_, R_2_ and R_3_ are the possible substituent groups. **(B)** An example of a catechin (+)-catechin. **(C)** General structure of a proanthocyanidin, where n indicates the number of repetitions of the monomer in brackets. The bond between the monomers is called an interflavan linkage.

Condensed tannins, or proanthocyanidins, are a highly diverse class of polymers and/or oligomers of a flavonoid called flavan-3-ol. Structurally speaking, flavanols are composed of a carbon backbone of C6-C3-C6, where the C6 compounds correspond to aromatic rings (A- and B-ring) and the C3 is a dihydropyran ring (C-ring) (Aron and Kennedy, 2008). The A-ring is similar to a resorcinol moiety and the B-ring is similar to a catechol moiety (Figure 2A). The monomers of flavan-3-ols are called catechins, and they differ among them in the stereochemistry of the C2 and C3, the presence or absence of galloyl groups, and the hydroxylation of the B-ring (Watrelot and Norton, 2020). When the substituents are R_1_ = R_3_ = H and R_2_ = OH, then the molecule is (+)-catechin (Figure 2B). As flavan-3-ols have two chiral carbons, they possess four diastereisomers, so one isomer of (+)-catechin (2*R*, 3*S* configuration) is (+)-epicatechin (2*S*, 3*S* configuration). In nature, the configuration 2*R* is more common than the 2*S* configuration (Aron and Kennedy, 2008).

Aron and Kennedy (2008) described thirteen types of proanthocyanidins based upon the hydroxylation of A-ring and B-ring carbons and the C3 from the C-ring. Hydroxylation of C8 (A-ring) is the most uncommon modification of proanthocyanidins, present only in the cases of proteracacinidin and promelacacinidin. By contrast, in the case of C3 (C-ring), only in 5 types a proton is present instead of a hydroxyl group (Aron and Kennedy, 2008). In this classification, it is not contemplated that C3 substituents despite hydroxyl groups.

### Proanthocyanidins Biosynthesis, Transport, and Accumulation

**FIGURE 3:**
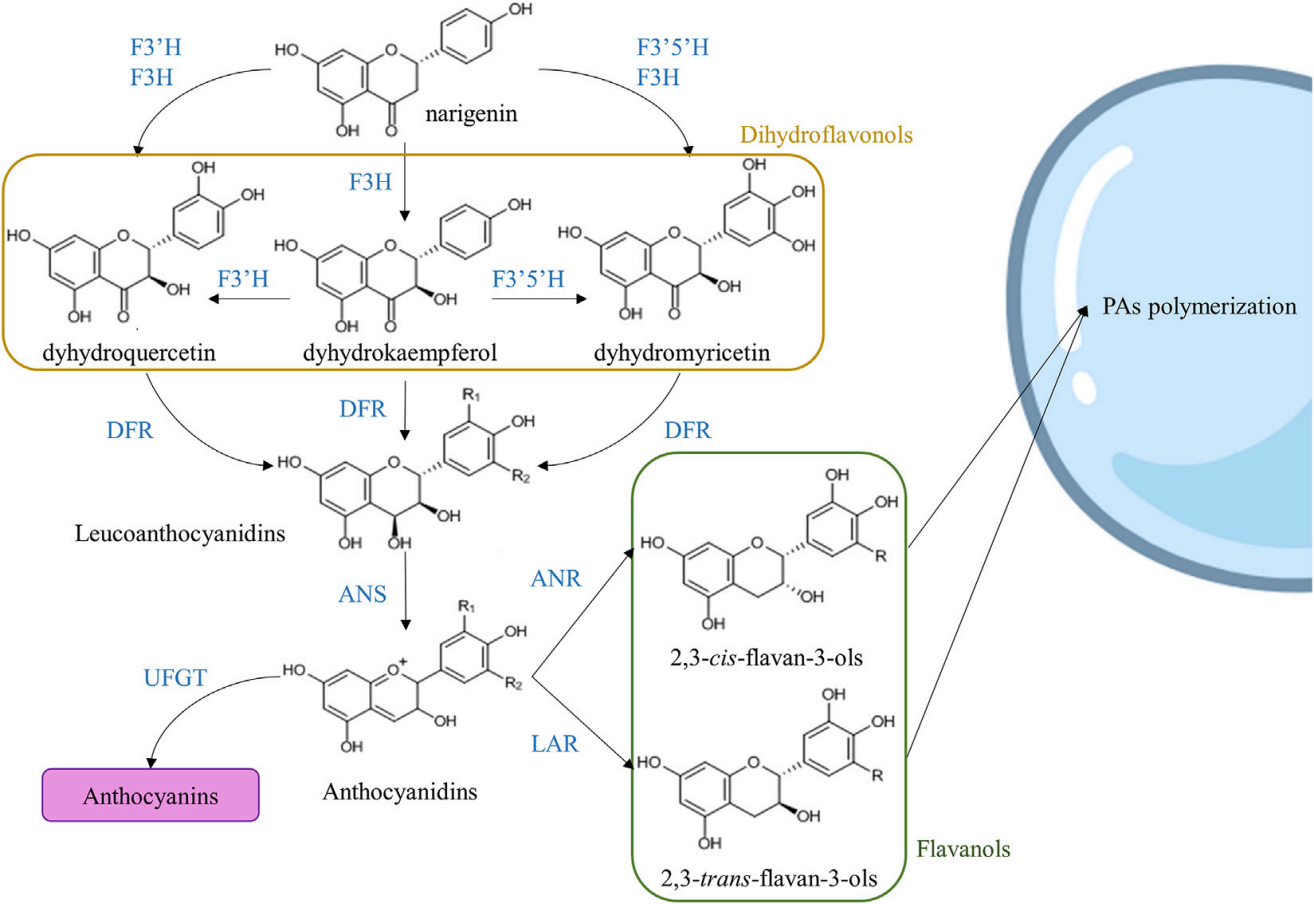
Simplified flavonoid pathway. One branch of this pathway yields flavanols that can be transported to the vacuole by several proposed mechanisms. Abbreviations: flavonoid 3-hydroxylase (F3H), flavonoid 3′-hydroxylase (F3′H), flavonoid 3′5′-hydroxylase (F3′5′H), dihydroflavonol 4-reductase (DFR), anthocyanidin synthase (ANS), UDP-glucose flavonoid 3-O-glucosyltransferase (UFGT), anthocyanidin reductase (ANR), and leucoanthocyanidin reductase (LAR). Modified from Akagi et al., 2011; Yu et al., 2020; Wei et al., 2021.

Proanthocyanidins are synthesized from L-phenylalanine through the phenylpropanoid and flavonoid pathways. First, L-phenylalanine is transformed into cinnamic acid or cinnamate by the phenylalanine ammonia lyase (PAL), and the cinnamate is hydroxylated to *p*-coumaric acid by means of cinnamate 4-hydroxylase (C4H). The next step is the addition of coenzyme A (CoA) to the *p*-coumaric acid by the catalysis of 4-coumaroyl-CoA ligase (4CL), yielding *p*-coumaroyl-CoA (Vogt, 2010; Deng and Lu, 2017). 4-coumaroyl-CoA and 3 molecules of malonyl-CoA are the substrates of chalcone synthase (CHS), which catalyzes the first step of the flavonoid pathway, yielding naringenin chalcone, and chalcone isomerase (CHI) then convertes this molecule to the flavanone naringenin. Naringenin serves as a substrate for flavonoid 3-hydroxylase (F3H), flavonoid 3′-hydroxylase (F3′H) and flavonoid 3′5′-hydroxylase (F3′5′H). If naringenin is the substrate of F3′5′H and F3H, the product is a dihydroflavonol called dihydromyricetin, while the combined action of F3′H and F3H yields the production of dihydroquercetin. Dihydroflavonol 4-reductase (DFR) converts dihydromyricetin to leucodelphinidin (R_1_ = R_2_ = OH in Figure 3) and dihydroquercetin to leucocyanidin (R_1_ = OH and R_2_ = H in Figure 3). Then, the antocyanidin synthase (ANS), also called leucoanthocyanidin dioxygenase (LDOX), produced delphinidin and cyanidin from leucodelphinidin and leucocyanidin, respectively. Additionally, dihydrokaempferol is derived from naringenin and can be converted into leucopelargonidin (R_1_ = R_2_ = H in Figure 3) and pelargonidin by DFR and ANS catalysis, respectively. Cyanidin, delphinidin, and pelargonidin can be transformed into 2,3-cis-flavan-3ols (epi)catechin, (epi)gallocatechin, and (epi)afzelechin, respectively), by the action of anthocyanidin reductase (ANR). On the other hand, 2,3-*trans*-flavan-3-ols are synthetized from leucocyanidin, leucodelphinidin, and leucopelargonidin by the action of leucoanthocyanidin reductase (LAR) (Figure 3) (Akagi et al., 2011; Yu et al., 2019; Wei et al., 2021).

PAs are found to accumulate exclusively in the vacuoles, forming glycoside conjugates (Grotewold, 2004; Kitamura et al., 2010; Fontes et al., 2011). In particular, some vacuoles specialized in PA accumulation are known as “tannin vacuoles” (Terrier et al., 2009). The knowledge about biophysical and biochemical characteristics of vacuoles’ membranes is very elusive, converting tannin transport into these organelles in a poorly known mechanism. One mechanism proposed for the transport of PAs from the cytosol to the vacuole is membrane transporters. There are three kinds of putative membrane transporters for PAs: ATP binding cassette (ABC) transporters, mammalian bilitranslocase (BLT) transporters, and multidrug detoxification and extrusion (MATE) transporters (Rousserie et al., 2019). Only ABC transporters act as primary active transporters, using ATP for transport, while BLT and MATE transporters function as secondary active transporters, using an electrochemical gradient to move PAs.

The name “condensed tannins” for PAs is because the acid hydrolysis of these compounds releases anthocyanidins due to the breakage of interflavan linkages (Deng and Lu, 2017). As mentioned before, PAs are built of flavan-3-ol monomers linked by carbon-carbon bonds (Dixon et al., 2013). Actual knowledge indicates that (+)-catechins and (-)-epicatechins are thought to be starter units for PA polymerization (Xie et al., 2003; Kitamura et al., 2004; Dixon et al., 2005). Due to the interflavan linkages, two types of PAs can be distinguished. The A-type consists of subunits linked by C4-C8 and/or C4-C6 (A-ring carbons) and C2-*O*-C7 or C2-*O*-C5 bonds. On the other hand, B-type PAs presents only the two possible linkages C4-C8 and/or C4-C6 (Yu et al., 2020). The distribution of A-type and B-type PAs is different in nature. A-type PAs are mainly found in berries like bilberry (*Vaccinium myrtillus*) and cranberry (*Vaccinium oxycoccus*), cinnamon (*Cinnamomum* spp.) and peanuts (*Arachis hypogaea*), for example (Gabetta et al., 2000; Gu et al., 2002; Gu et al., 2003), while B-types are widely distributed (Gabetta et al., 2000; Gu et al., 2003; Suvanto et al., 2020). Condensed tannins are responsible for the bitterness and astringency of many fruits. For example, in apples, astringency is correlated to the mean degree of polymerization (mDP) and the galloylation degree of these compounds, but it is negatively correlated with the hydroxylation level of the B-ring of flavan-3-ols (Saxe et al., 2021).

In the next sections, we focus on the genetic factors known so far to be involved in PAs regulation, which are summarized in Table 1.

**TABLE 1:** Summary of principal genes regulating tannins biosynthesis in crops species.

Gene	Gene name	Species	References
Condensed tannins or proanthocyanidins			
*DkANR*	*Anthocyanidin reductase*	*Diospyros kaki*	Ikegami et al. (2007)
*DkLAC1*	*Laccase*	*Diospyros kaki*	Mo et al. (2015)
*DkLAR*	*Leucoanthocyanidin reductase*	*Diospyros kaki*	Ikegami et al. (2007)
*DkMYB2/4*	*MYB-family transcription factor*	*Diospyros kaki*	Akagi et al. (2009b), Akagi et al. (2010a)
*DkMYC1*	*bHLH-family transcription factor*	*Diospyros kaki*	Naval et al. (2016)
*FabHLH3*	*bHLH-family transcription factor*	*Fragaria* x *ananassa*	Schaart et al. (2013)
*FaMYB9/11*	*MYB-family transcription factor*	*Fragaria* x *ananassa*	Schaart et al. (2013)
*MdMYB9/11*	*MYB-family transcription factor*	*Malus domestica*	Sun et al. (2019)
*MdMYBPA1*	*MYB-family transcription factor*	*Malus domestica*	Wang et al. (2018)
*PpMYB7*	*MYB-family transcription factor*	*Prunus persica*	Zhou M. et al. (2015)
*PpMYB18*	*MYB-family transcription factor*	*Prunus persica*	Zhou et al. (2019)
*VvLAR1/2*	*Leucoanthocyanidin reductase*	*Vitis vinifera*	Bogs et al. (2005)
*VvMYB5a/5b*	*MYB-family transcription factor*	*Vitis vinifera*	Deluc et al. (2006), Deluc et al. (2008)
*VvMYBC2-L1/2/3*	*MYB-family transcription factor*	*Vitis vinifera*	Yu et al. (2020)
*VvMYBPA1*	*MYB-family transcription factor*	*Vitis vinifera*	Huang et al. (2014)
*VvMYBPA2*	*MYB-family transcription factor*	*Vitis vinifera*	Terrier et al. (2009)
Hydrolysable tannins			
*CsSDH*	*Shikimate dehydrogenase*	*Camellia sinensis*	Huang et al. (2014)
*DkSDH*	*Shikimate dehydrogenase*	*Diospyros kaki*	Akagi et al. (2009a)
*FaTA*	*Tannase*	*Fragaria* x *ananassa*	Dai et al. (2020)
*PgSDH3s/4*	*Shikimate dehydrogenase*	*Punica granatum*	Habashi et al. (2019)
*PgUGT84A23/24*	*UDP-glycosyltransferase*	*Punica granatum*	Ono et al. (2016)
*VvSDH3/4*	*Shikimate dehydrogenase*	*Vitis vinifera*	Bontpart et al. (2016)

### Proanthocyanidin Regulation

**FIGURE 4:**
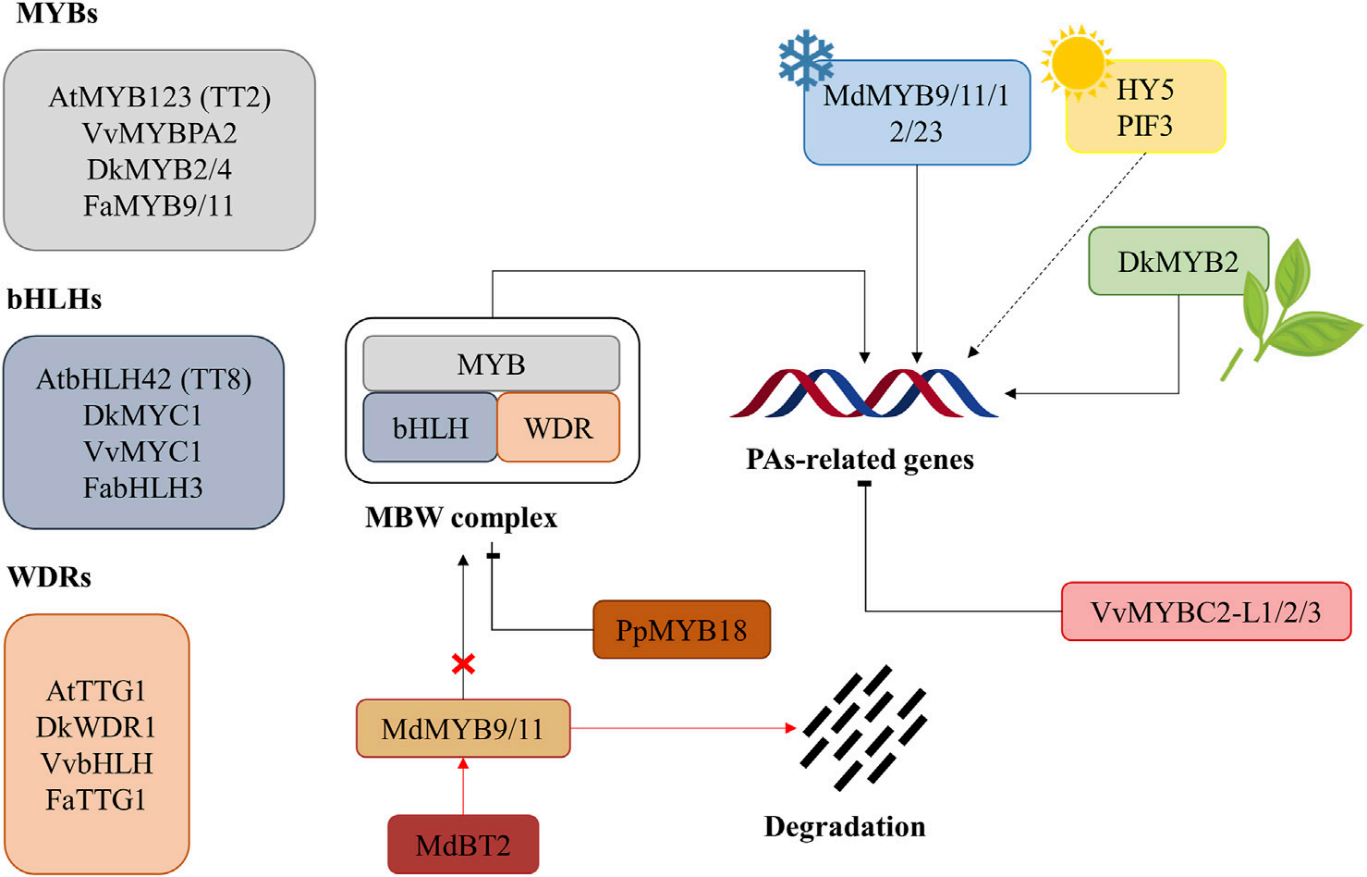
General regulation scheme of proanthocyanidin synthesis. The scheme shows regulation via MBW-complex formation and cold-, light- and wounding-induced regulation. The degradation of MdMYB9/11 through BT2 and the proteasome is also shown with the red arrows, resulting in the impossibility of these two transcription factors to form the MBW-complex. Arrows indicate positive regulation, while flat-ended lines mean negative regulation (repression). Dotted lines indicate a process not fully elucidated. At: *Arabidopsis thaliana*, Dk: *Diospyros kaki*, Fa: *Fragaria* x *ananassa*, Md: *Malus domestica*, Pp: *Prunus persica*, Vv: *Vitis vinifera*.

Regulation of proanthocyanidin biosynthesis occurs mainly at the transcriptional level, especially for the genes encoding biosynthetic enzymes (Koes et al., 2005; Xu et al., 2015). PAs synthesis is regulated during fruit growth, as their accumulation starts with the development of the fruit and stops when it starts ripening (Wei et al., 2021). The regulation of PAs is well-known in *A. thaliana* where there are 6 families of transcription factors thought to participate in PAs synthesis regulation: *MYB*, *basic helix-loop-helix* (*bHLH*, also known as *MYC*), *tryptophan-aspartic acid repeat (WDR)-containing protein 40 (WD40), WRKY, and MADS-box proteins and zinc fingers* (Terrier et al., 2009; Rousserie et al., 2019). The MYB-bHLH-WD40 (MBW) complexes are essential regulator elements for PAs accumulation and gene expression (Figure 4; Table 1) (Koes et al., 2005; Ramsay and Glover, 2005; Wei et al., 2021). Due to the ubiquity of *bHLH* and *WD40* in transcriptional regulation, it is the C-terminal part of the MYB protein in the MBW-complex that is responsible for the specificity in activating target genes. Meanwhile, *bHLH* is responsible for binding to the nucleotide sequence (normally E-box or G-box *cis*-elements) (Xu et al., 2014), and WD40 is crucial for the stabilization of the complex (Huang et al., 2014; Yu et al., 2020). Both transcription factors, *MYB* and *bHLH*, can specifically interact with nucleotide sequences within the flavonoid promoter. MYBCORE and AC-rich elements are the two main binding sites for *MYBs* in the regulation of flavonoids and PAs, although the exact motif for *MYB* binding remains unknown in most PAs structural genes (Constabel, 2018). In *A. thaliana,* the MBW-complex is composed of *AtMYB123* (or *Transparent Testa, TT2*), *AtbHLH42* (or *TT8*), and *Transparent Testa Glabra31* (or *TTG3*), which is the WD40 protein. This complex is responsible for the activation of *ANR* (Rousserie et al., 2019), *LDOX* and *DFR* genes, leading to the accumulation of PAs in the seed coat (Nesi et al., 2001; Baudry et al., 2004). In particular, TTG2-like proteins are thought to be crucial in PA transportation (Debeaujon et al., 2001; Gonzalez et al., 2016). Homologs of *TT2*, *TT8,* and *TTG1* in other fruits and plants are often searched for their role in flavonoid and PA regulation (Yu et al., 2020).

Besides regulation at the gene level, the biosynthesis of PAs is affected by biotic and abiotic stresses and plant hormones. Environmental or abiotic stresses, such as low temperature, drought, or wounding, among others, may lead to the production of ROS and the consequent oxidative stress responses (Suzuki et al., 2012). As described previously, flavonoids and other antioxidant compounds can palliate the action of ROS, and the genes responsible for their synthesis can be induced under these circumstances (Winkel-Shirley, 2002). Water quantity has been proven to influence the phenolic content of grapes (Castellarin et al., 2007). Light also plays a part in the regulation of PAs, and it has been suggested that light-signaling proteins, such as HY5 and PIF3, may interact with some promoters of PAs-related genes, like *CHS*, *CHI,* and *DFR* (Shin et al., 2007). The action of plant hormones courses through their interaction with MBW-complexes, thus regulating flavonoid genes at the transcriptional or post-transcriptional level (Jaakola, 2013). In addition to abiotic stresses, plants are also exposed to the influence of fungi, bacteria, viruses, and herbivores. In this context, accumulation of PAs is a plant defense strategy against these biotic stresses (Dixon et al., 2005; Ullah et al., 2017). The two main hormones related to biotic stresses are salicylic and jasmonic acids, suggesting a possible role for both molecules in PA regulation.

### Proanthocyanidins in Persimmon Fruit (*Diospyros kaki*) and Its Regulation

Persimmon possesses PAs-rich fruits which make them inedible until physiological ripening or by artificial treatment, such as ethanol. In persimmons, PAs monomers are mainly prodelphinidins and they present a high degree of galloyl residues (Li et al., 2010). *Pollination constant and non-astringent* (PCNA) is a persimmon mutant spontaneously generated, whose principal characteristic is the total absence of astringency, making it a desirable fruit for consumption. The absence of astringency is explained by the reduced accumulation of epigallocatechin and epigallocatechin gallates at the early stage of fruit development.

In persimmon, several PAs-biosynthesis related genes have been identified: *ANR* (*DkANR*) and *LAR* (*DkLAR*) (Ikegami et al., 2007; Nakagawa et al., 2008; Akagi et al., 2009b). *In vitro* assays of *DkANR* in *Escherichia coli* showed a conversion of anthocyanidins to both isomers 2,3-*cis*-flavan-3-ols and 2,3-*trans*-flavan-3-ols (Akagi et al., 2009a), demonstrating the role of *DkANR* in PA accumulation in persimmon fruit. However, further steps in PA biosynthesis in persimmon are poorly known (Zhao et al., 2010), with the exception of *DkLAC1*, a laccase gene that participates in the polymerization of PAs (Mo et al., 2015). Several transcription factors crucial for PA accumulation in persimmon have been identified as *DkMYB2/4*, *DkMYC1,* and *DkWDR1* as homologs of *TT2*, *TT8,* and *TTG1*, respectively (Akagi et al., 2009b; Akagi et al., 2010a; Naval et al., 2016). In yeast and *Nicotiana benthamiana,* it has been shown that *DkMYB2, Shalini,* and *DkWDR1* interact together to act as a MBW-complex (Gil-Muñoz et al., 2020). Moreover, in PCNA fruits, *Dk*MYB4 regulates the expression of *ANR* and *F3′5′H* (Akagi et al., 2009b). *DkMYB4* is expressed mainly in fruit flesh and seed during development, and it was shown to bind to MYBCORE *cis*-motifs of the promoter region of three PAs-biosynthetic genes (Figure 3): *DkANS, DkF3′5′H,* and *DkANR* (Akagi et al., 2009b). However, in the same study, *Dk*MYB4 was shown not to regulate the expression of *DkLAR* (Akagi et al., 2009b), supporting the possibility that in persimmon, like in other species, the expression level of *LAR* did not correlate with PA accumulation (Pang et al., 2007). Overexpression of *DkMYB4* results in accumulation of PAs with toxic effects when expressed ectopically in persimmon and kiwifruit (*Actinida deliciosa*) calli (Akagi et al., 2009b), while RNAi knockdown results in a similar expression pattern to the one observed in PCNA fruits (Dixon et al., 2013). Of interest, *DkMYB4* expression was in turn regulated by *Dk*b*ZIP5*, an abscisic acid (ABA)-responsive transcription factor (Akagi et al., 2012). The presence of ABA-responsive elements in the *DkMYB4* promoter suggests that the phytohormone ABA may modulate PA biosynthesis and content (Akagi et al., 2012).

Another *MYB* gene has been described as playing a part in the regulation of PAs. *DkMYB2* is a wound-inducible expression factor (see Figure 4) whose ectopic expression in persimmon and kiwifruit calli positively regulates the expression of *ANR* and *LAR* (Akagi et al., 2010a). Another interesting point is the regulation of PAs pathway genes by the action of ethanol treatment in order to remove astringency (Araki et al., 1975; Ikegami et al., 2007). Plants treated with ethanol (contained in plastic bags with 10 ml of 5% ethanol) (Ikegami et al., 2007) presented lower expression of two shikimate pathway genes, most PA pathway genes, and serin carboxypeptidase-like proteins, that are putatively involved in PA accumulation (Terrier et al., 2009), compared to the control non-treated plants (Ikegami et al., 2007). In this sense, the hypothesis of astringency removal is that ethanol both reduces PA biosynthesis and enhances PA solubility (Akagi et al., 2011).

### Proanthocyanidins in Grapevine (*Vitis vinifera*) and Wine

Condensed tannins are highly studied in the case of grapes and wine due to their ability to offer astringency and change the organoleptic properties of the wine (Robinson et al., 2021). PAs are found mostly in red wines, a fact caused by maceration and skin contact, which does not occur in white wines. During alcoholic fermentation, both PAs extraction and mDP of tannins increase (Aron and Kennedy, 2008). In grapes, skin PAs are different from seeds PAs not only in their composition, but also in the mDP. Skin PAs are catechin-rich polymers, with epicatechin and epigallocatechin being the most common terminal units, and mDP being around 31–33, depending on the variety (Souquet et al., 1996; Hanlin and Downey, 2009). Seeds PAs are rich in epicatechin either as a monomer or as an extension unit (Dixon et al., 2013), and the mDP varies depending on the variety: 3.8–5.9 in Cabernet Sauvignon and 8–11 in Syrah before and after harvest maturity (Kyraleou et al., 2017; Blancquaert et al., 2019; Watrelot and Norton, 2020).

For *V. vinifera,* much more is known in the case of MYBs factors than in those of bHLH and WD proteins. In the case of bHLHs, VvMYC1 is thought to activate *VvCHI* and *VvANR* promoters by interacting with VvMYBPA1 (Huang et al., 2014). Indeed, it is required the interaction between VvMYC1 and VvMYBPA1 for the activation of the two promoters, leaving in evidence that VvMYC1 by its own cannot trigger PAs biosynthesis (Hichri et al., 2010). *VvMYB5a* and VvMYB5b were discovered to regulate positively PAs synthesis in skin, flesh and seeds of grapes during the early stages of fruit development (Deluc et al., 2006; Deluc et al., 2008) and they have been reported to regulate promoters of the flavonoid genes *VvCHI* and *VvLAR1* together with *AtEGL3* (*bHLH* partner). *VvMYB5b* overexpression in tobacco (*Nicotiana tabacum*) induced a rising in PAs concentration (Deluc et al., 2006; Deluc et al., 2008). On the other hand, overexpression of *VvMYB5a* caused a strong increase in several metabolites of the phenylpropanoid pathway, including anthocyanins, flavonols, and PAs, as well as an alteration in lignin metabolism, suggesting that this transcription factor may be involved in the regulation of the different branches of this pathway.

Interestingly, in *V. vinifera,* the transformation of 3-deoxy-leucocyanidin is controlled by two highly related genes: *VvLAR1* and *VvLAR2* (Bogs et al., 2005; Wei et al., 2021). Curiously, VvMYBPA1 and VvMYBPA2, another MYB factor identified in grapes, can activate *ANR* and *LAR1*, but not *LAR2*, affecting directly the flavonoid pathway (Bogs et al., 2007; Terrier et al., 2009; Wei et al., 2021). Silencing of *ANR* genes in *V. vinifera* resulted in an accumulation of anthocyanins and flavonols as a consequence of metabolic flux redirection towards other flavonoid compounds rather than proanthocyanidins (see Figure 3) (Robinson et al., 2021). Paolocci et al. (2007) demonstrated that *ANR* and *LAR1* are regulated by the same bHLH factor that enhances the accumulation of PAs. Furthermore, the expression pattern of *VvLAR1* and *VvLAR2* is suggested to differ in skin and seed grapes, with *VvLAR1* being specific of seed, while *VvLAR2* can be found in both tissues (Wei et al., 2021). In addition, the *LAR* gene has been reported to be involved in determining tannin polymer length; indeed, when this gene is knocked-out, grape seed PAs have higher mDP in transgenic lines (Robinson et al., 2021). In addition, the expression of *VvWRKY26* (a member of the *WRKY* family) in grape tissues participates in the regulation of PAs biosynthesis and vacuolar transportation (Wei et al., 2021).

On the other hand, several repressors of PA biosynthesis have been identified, such *asVvMYBC2-L1, VvMYBC2-L2 or VvMYBC2-L3* (Yu et al., 2020). Overexpression of both *VvMYBC2-L1* and *VvMYBC2-L3* in grapevine hairy roots results in a significant decrease in PAs concentration. Interestingly, some microRNAs (miRNAs) may be important in the regulation of PAs and polyphenol biosynthesis (Rock, 2013). The miRNA TAS4/miR828 targets *VvMYBA6* and *VvMYBA7*, two genes with homology with*VvMYBPA1* and *VvMYBPA2* (Rock, 2013). These repressors cause a reduction in the transcription of flavonoid and phenylpropanoid structural genes in addition to the inhibition of PA biosynthesis, as they also disrupt the transcriptional activation of the MBW complex (Yoshida et al., 2015; Ma et al., 2018).

Water conditions are determinant in grape characteristics, in fact water deficit can regulate the genes involved in the biosynthesis of PAs (Casassa et al., 2015). Full irrigated *V. vinifera* showed grape skin and seed extracts with high astringency with respect to non-irrigated plants (Kyraleou et al., 2016). One hypothesis is that water deficits may induce *VvLAR2* and *VvMYBPA1*, increasing PAs levels and their polymerization in the Cabernet Sauvignon variety (Yu et al., 2020). Light can also affect gene expression; one example is that *VvDFR* expression, needed for the synthesis of PAs precursors (Figure 3), can be induced by light (Gollop, 2002; Wei et al., 2021). In addition, light can trigger the transcription of *VvCHS2*, *VvDFR,* and *VvLDOX* compared with shading treatment (Liu W. et al., 2017; Wei et al., 2021). On the other hand, the effect of temperature on gene expression is not conclusive because some studies do not find any correlation between high temperature and PAs concentration (Mori et al., 2004; Pastore et al., 2017), but some others show that high temperature causes PAs accumulation in grapes (Bonada et al., 2015; Poudel et al., 2020), due to a decrease in the levels of *VvANR* and *VvLAR1* mRNA. Additionally, *VvMYBA1* mRNA accumulated when treated with ABA, leading to increased levels of PAs but also higher levels of anthocyanins and PAs structural genes (Jeong et al., 2004).

### Proanthocyanidins Regulations in Other Crops

Other crops have been the object of study in what pertains to PAs regulation. In reference to strawberries (*Fragaria* x *ananassa*), the major and common compounds related to PAs are catechin, proanthocyanidin B1, proanthocyanidin trimer, and proanthocyanidin B3 (Giampieri et al., 2012; Fierascu et al., 2020). In strawberry, epicatechins and catechins levels, as well as procyanidin levels, decrease with ripening (Aaby et al., 2012), specifically in the final maturation stage, this tendency has been observed in other species like bilberry (Suvanto et al., 2020). The functional orthologues of *AtTT2*, *AtTT8,* and *AtTTG1* have been elucidated in strawberry, being *FaMYB9/FaMYB11*, *FabHLH3,* and *FaTTG1*, respectively (Schaart et al., 2013). In a yeast-two hybrid assay, *FaMYC1* and *FabHLH33* were also discovered to be related to PA synthesis, but their implication remains elusive. It is speculated that FaMYC1 and FabHLH33 could dimerize in the same way as other bHLHs (Feller et al., 2006). Derived from the same work, Schaart et al. (2013) hypothesized that *FaMYB5* and *FabHLH3Δ*, a truncated version of *FabHLH3* identified in strawberries, act as negative regulators of flavonoid biosynthesis. *FaMYB5* may combine with *FaMYB1*, a confirmed repressor of PAs biosynthesis with high sequence homology to *VvMYBC2-L1* (Aharoni et al., 2001; Paolocci et al., 2011), and they together interfere with the activity of MBW-complexes. On its part, FabHLH3Δ is capable of interacting with FaMYB9 or FaMYB11 and causing the blockage of their binding sites for bHLHs, avoiding the MBW-complex formation (Schaart et al., 2013). Interestingly, it was found that *FaTT12-1* is a putative orthologue of *AtTT12*, which is light-sensitive. In fact red light can significantly activate its expression (Chen et al., 2018), suggesting a possible role in PA biosynthesis regulation. Delgado et al. (2018) demonstrated that treatment with methyl jasmonate caused upregulation of *FaANS*, *FaLAR,* and *FaUFGT* genes and a downregulation of the *FaANR* gene compared to the control, leading to increased anthocyanin levels and decreased PAs content. On the other hand, treatment with methyl jasmonate and jarin-1, an inhibitor of the bioactive form of jasmonate, jasmonoyl-isoleucine, caused the opposite effect. The increase of PAs in fruits treated with methyl jasmonate and jarin-1 could be explained by the upregulation of *FaMYB9*, *FaMYB11,* and *FabHLH33* together with *FaANR* (Delgado et al., 2018). Together, these data suggest a connection between the phytohormone jasmonic acid and PA biosynthesis during strawberry fruit ripening. Inoculation experiments with *Colletotrichum acutatum* and *Botrytis cinerea* in strawberries at different maturity stages showed that fungal infection was much higher in the case of riper fruits, but almost absent in the case of unripe strawberries, where the expression levels of *LAR* and *ANR* were found to be higher (Guidarelli et al., 2011; Haile et al., 2019). This matches with the fact that flavan-3-ols and PA concentrations decrease as ripening progresses (Carbone et al., 2009; Nagpala et al., 2016), reinforcing the role of PAs in plant defense against biotic stresses.

The *MYBPA1* ortholog in apple (*Malus domestica*) is *MdMYBPA1* (Wang et al., 2018) found that PAs are not only regulated through the MBW-complex in this crop; indeed, a NAC transcription factor, NAC52, has been recently reported for its ability to bind to the promoters of *MdLAR, MdMYB9,* and *MdMYB11*, and this union leads to PAs accumulation when *MdNAC52* is ectopically overexpressed (Sun et al., 2019). Interestingly, a negative regulation of PAs in apples occurs via the degradation of MdMYB9 protein by MdBT2, which substantially reduces PAs concentration (An et al., 2018). MdBT2 is a bric-à-brac, tramtrack, and broad complex protein, essential for the degradation of proteins through the ubiquitin proteasome system. Low temperatures trigger the expression of *MdMYB9/11/12/23* and *MdMYBPA1*, a series of transcription factors capable of binding promoters of PAs structural genes, causing a rise in PAs levels, hypothetically to prevent damage due to cold stress (Gesell et al., 2014; An et al., 2018; Wang et al., 2018). MdMYB23 protein is induced by cold stress, which leads to PAs accumulation, but as MdMYB9, it is normally degraded by MdBT2 via ubiquitin-proteasome pathway (An et al., 2018). Jasmonic acid is reported to increase PA accumulation due to an upregulation of *MdMYB9* and *MdMYB11* (An et al., 2015). MdJAZ18 is a repressor of the jasmonate signaling pathway that is susceptible to phosphorylation by MdSnRK1.1. Once phosphorylated, MdHAZ18 is degraded by the 26 S proteasome, causing the release of MdbHLH3. MdbHLH3 can then act with MdMYB9 and MdMYB11 to increase anthocyanins and PA synthesis (Liu X.-J. et al., 2017). In peach (*Prunus persica*), PpMYB7 is reported to bind to the promoter of *PpLAR*, thus regulating PA synthesis (Zhou H. et al., 2015). A R2R3-MYB repressor, *PpMYB18*, downregulates PAs biosynthesis in transgenic tobacco (*Nicotiana tabacum*) leaves (Zhou et al., 2019). Interestingly, this transcription factor can be induced by PAs-related MYB activators (*PpMYBA1* and *PpMYB10.1*), suggesting a role in the fine-tuning of the levels of these important metabolites by providing a feedback regulation of the MBW-complex during fruit ripening. *PpMYB18* may act as both an active and passive repressor of PA biosynthesis by competing with PpMYB10.1 and PpMYBPA1 for the union with PpbHLHs (Zhou et al., 2019).

## Hydrolysable Tannins: Ellagitannins and Gallotannins

The presence of hydrolysable tannins in food and beverages is more uncommon than the presence of condensed tannins. They can be found in black currant (*Riges nigrum*), blackberries (*Rubus fructicosus*), strawberries, raspberries (*Rubus occidentalis*), and pomegranate (*Punica granatum*), guava (*Pisidium spp.*), mango (*Magnifera indica*) and in nuts like almonds (*Prunus dulcis*), pecans (*Carya illinoinensi*s), and walnuts (*Juglans regia*), with the total absence in species like legumes and cereal grains (Landete, 2011; Smeriglio et al., 2017).

**FIGURE 5:**
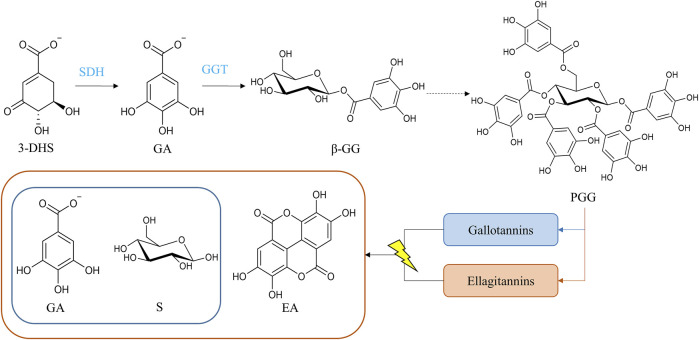
Biosynthetic pathway of hydrolysable tannins. The hydrolysis of gallotannins and ellagitannins yields gallic acid and sugar (glucose represented) and gallic acid, ellagic acid, and sugar, respectively. Dotted lines represent multistep reactions. Abbreviations: 3-dehydroshikimate (3-DHS), gallic acid (GA), sugar unit (S), ellagic acid (EA), β-glucogallin (β-GG), pentagalloylglucose (PGG), shikimate dehydrogenase (SDH), and β-glucogallin-dependent galloyltransferases (GGT).

On the opposite of proanthocyanidins, much less is known regarding hydrolysable tannins synthesis and regulation. Structurally, hydrolysable tannins are based on a central sugar core, mainly β-D-glucose, in which phenolic compounds are esterified through its hydroxyl groups (Grundhöfer et al., 2001; Quideau et al., 2011). There are two major classes of these compounds depending on the phenolic groups: gallotannins (gallic acid) and ellagitannins (hexahydroxydiphenic acid, HHDP) (Bar-Ya’akov et al., 2019). Acid hydrolysis of gallotannins yields sugar and gallic acid, whereas the hydrolysis of ellagitannins provides sugar, gallic acid, and ellagic acid (Figure 5) (Smeriglio et al., 2017). Gallotannins are the simplest hydrolysable tannins and are rarely found in nature. They are present in some woody species like mango and almonds; on the other hand, ellagitannins are more common in fruits, nuts and seeds (Landete, 2011; Smeriglio et al., 2017). In fact, a myriad of ellagitannin compounds have been identified to date, principally due to their ability to oligomerize (Okuda and Ito, 2011).

The key molecule for hydrolysable tannin biosynthesis is gallic acid, which is derived from 3-dehydroshikimate via a shikimate dehydrogenase (Niemetz and Gross, 2005; Muir et al., 2011; Bontpart et al., 2016). Furthermore, gallic acid is relevant for the formation of epigallocatechin and epicatechin gallate (Liu et al., 2012), which are proanthocyanidin monomers. Downregulation of the shikimate dehydrogenase, *DkSDH,* in persimmon resulted in a decrease in epigallocatechin content in PCNA-type mutants (Akagi et al., 2009a), which matches the implication of gallic acid in both types of tannins. Studies in *Camellia sinensis* and *V. vinifera* indicate the role of shikimate dehydrogenase in generating gallic acid (Bontpart et al., 2016; Huang et al., 2019). Gallic acid is then esterified with UDP-glucose (Gross, 1983), to form 1-*O*-galloyl-β-D-glucopyranose (also known as β-glucogallin), which is the simplest gallotannin. Ono et al. (2016) found two UDP-glycosyltransferases (UGTs) in *P. granatum*, PgUGT84A23 and PgUGHT84A24, related to β-glucogallin synthesis. According to studies in oak (*Quercus rubur* and *Quercus rubra*) and sumac (*Rhus typhina*), the next steps of galloylation occur with a remarkable specificity following the sequence: β-glucogallin, 1,6-digalloylglucose, 1,2,3-trigalloylglucose, 1,2,3,6-tetragalloylglucose and finally, 1,2,3,4,6-pentagalloyglucose (Schmidt et al., 1987; Gross and Denzel, 1991). These subsequent additions of galloyl moieties (Haslam and Cai, 1994) result in the formation of 1,2,3,4,6-pentagalloyl-D-glucose, the common precursor of both gallotannins and ellagitannins (Figure 5). Pentagalloylglucose can be oxidized to generate 3,4,5,3′,4′,5′-hexahydroxyphenoyl (HHDP) residues, a molecule that once liberated from the sugar core in the form of hexahydroxydiphenic acid, spontaneously lactonizes into ellagic acid (Niemetz and Gross, 2005; Smeriglio et al., 2017). The oxidation of pentagalloylglucose occurs via oxygen-dependent laccase-type enzyme catalyzation in *Tellima grandiflora* (Niemetz et al., 2001; Niemetz et al., 2003; Niemetz and Gross, 2003a; Niemetz and Gross, 2003b).

Besides β-D-glucose, which is by far the most common sugar core for gallotannins and ellagitannins, other exotic polyols may appear, such as xylose, sorbitol, fructose, or even not-sugar compounds like shikimic, and quinic acids. However, their abundance in nature is extraordinarily low and they are only found in maple (*Acer negundo*), chestnut (*Castanea sativa*) and witch-hazel (*Hamamelis* sp.) (Smeriglio et al., 2017). Galloylation reactions with β-glucogallin as substrate can yield a wide range of molecules from di-to octagalloylglucoses, although the most common is 1,2,3,4,6-pentagalloyl-D-glucose (Smeriglio et al., 2017). The enzyme β-glucogallin-dependent galloyltransferases (Niemetz and Gross, 2005) is thought to be involved in the modification of gallotannins, one example is the transformation of hexa-/heptagalloylglucoses into 3-*O*-trigalloyl-1,2,4,6-*O*-tetragalloyl-β-D-glucopyranose, and other gallotannins with high degree of galloylation (Niemetz and Gross, 2005). Two galloyl residues can form what is called a *meta*- or *para-*depside bond, which is the result of the esterification between the carbonyl group of gallic acid and the aromatic *meta*- or *para*-hydroxyl group and comprises one of the most common modifications of gallotannins (Smeriglio et al., 2017).

Ellagitannins can be present in monomeric forms (e.g. casuarticin, corilagin, castalagin, eugeniin, geraniin, corilagin, galloyl-HDDP-glucose, and potentillin), dimeric forms (e.g. sanguiin H-2, sanguiin H-6, agrimoniin, and lambertianin A), trimeric forms (e.g. nupharin C, nupharin E) or even oligomeric forms (e.g. hirtellin A). Ellagic acid is one of the most important hydrolysable tannins in *F. x ananassa* fruits (Ariza et al., 2018). It has been recently found that agrimoniin, which is a dimeric ellagitannin composed of two units of potentillin linked by a dehydrogalloyl group (Karlińska et al., 2021), is highly present in “Camino Real,” “San Andreas,” and “Festival” strawberry cultivars (Villamil-Galindo et al., 2021). In fact, agrimoniin has been proposed as a taxonomic marker for the *Rosaceae* family, to which strawberries belong (Grochowski et al., 2017). Other common ellagitannins in the *Fragaria* genus are sanguiin H-6, galloyl-bis-HHDP-glucose, and lambertianin C (Giampieri et al., 2012).

The information available in the literature about the genes involved in hydrolysable tannin synthesis is summarized in the next paragraphs and in Table 1.

Regarding ellagitannin biosynthesis, formation of HHDP through the C4-C6 bonding of two respective gallic acids leads to the synthesis of tellimagrandin II as the first product of the pathway in a reaction catalyzed by a laccase-type polyphenol oxidase, as described in strawberry (Karlińska et al., 2021). Esterification of two more gallic acids at C2 and C3 and a possible de-esterification in C1 of tellimagrandin II result in several monomers: pedunculagin, potentillin and casuartictin (Niemetz and Gross, 2005; Karlińska et al., 2021). Further steps implicate dimerization or condensation reactions, forming 3 types of C-*O*-C in the case of the *Roseaceae* family: GOD, DOG, and GOG, depending on the donor and the acceptor of the reaction. The GOG-type bond implies two galloyl groups, the DOG-type bond (most frequently found in oligomers) requires a hydroxyl group from an HHDP as donor and a galloyl group as acceptor, and finally, the GOD-type bond is created from a galloyl hydroxyl donor to form an ether linkage with a HDDP as acceptor (Quideau, 2009).

Pomegranate fruit is another example of a reservoir of hydrolysable tannins, with more than 30 chemical species identified in the fruit peel, juice, and seeds (Fischer et al., 2011; Mena et al., 2012; Ito et al., 2014; Ambigaipalan et al., 2017; Wu and Tian, 2017; Liu and Seeram, 2018). The most representative ellagitannins in pomegranate fruit peel are α- and β-punicalagin (Seeram et al., 2005), and gallagic acid, punicalagin, and ellagic acid glycosides count as hydrolysable tannins present in both juice and fruit peel. Only 3,3′-di-*O*-methylellagic acid and 3,3′,4′-tri-*O*-methylellagic acid are present exclusively in the seeds (Bar-Ya’akov et al., 2019). Once again, the accumulation of tannins can be modified by regulating the expression of genes encoding key enzymes in their biosynthesis. Under osmotic stress, two shikimate dehydrogenase transcripts, *PgSDH3s* and *PgSDH4*, were accumulated, which is thought to increase hydrolysable tannin concentration (Habashi et al., 2019). Moreover, Habashi et al. (2019) reported that sucrose and red light stress could affect the accumulation of gallic acid and hydrolysable tannins with the change of *SDH* expression in pomegranates.

Tannases are a family of enzymes also known as tannin acyl-hydrolases (EC 3.1.1.20) capable of breaking carboxylic ester bonds present in gallotannins and galloylated flavanols (Ramírez et al., 2008; Rodríguez et al., 2009). These enzymes have been extensively studied in bacteria and fungi (Banerjee et al., 2012), but there is still much to learn in plants. The work of Dai et al. (2020) revealed that transient overexpression and RNAi of a strawberry tannase (*FaTA*) provoke an alteration in ellagic acid content; in addition, several enzymes belonging to the class I carboxylesterase clade were identified in *V. vinifera*, *J. regia*, *Citrus clementine, D. kaki,* and *C. sinensis*, indicating the hypothetical existence of more tannase genes in plants. A recent work in *Juglandaceae* (Wang et al., 2021) reveals regulatory *cis*-elements found in tannase promoters: E-box and ARR1AT, involved in brassinolide and cytokine responsiveness, respectively, and W-box and WUN-motifs implicated in wound abiotic stress responses. Also, motifs related to flavonoid biosynthesis (MYB-related motifs) and MYC motifs, related to cell growth, were found in tannases from Chinese hickory (*Carya cathayensis*) and pecan (*Carya illinoinensis*). These last results suggest the possibility of studying the regulation of tannases belonging to the same clad described by Dai et al. (2020) in other species.

## Future Perspectives in Metabolic Engineering of Crops Regarding Tannins

The role of tannins in providing health benefits has been extensively studied (Lu et al., 2021), a reason for which there is an increasing interest in selecting crops or varieties with enhanced levels of these therapeutic compounds. However, as tannins also participate in the organoleptic characteristics of foods and beverages by adding attributes of astringency and bitterness, precaution needs to be taken in order to maintain fruit palatability. As discussed in this review, several strategies can be followed to achieve fruit deastringency, such as ethanol treatment. Nevertheless, there is a need to increase our knowledge about how astringency and bitterness are perceived. In addition, an interesting way of decreasing crop astringency would be to reduce the ratio between high molecular weight and low molecular weight tannins. In this sense, a more precise insight of the molecular mechanisms underlying tannin synthesis may pave the way to develop new varieties more flavorsome. Further research will allow us to achieve foodstuffs that combine higher levels of these health-promoting compounds with a grade of astringency that makes them edible for the consumer. This could be the subject of genetic and biochemical regulation, taking into account the principal regulator genes of tannin biosynthetic pathways. The application of CRISPR/Cas9 technologies could be a solution in the improvement of new fruits by editing genes related to tannins (Zhang et al., 2020). These strategies can be coupled to genome-wide association studies (GWAS) or QTL mapping to find genes, alleles, or markers associated with a high content of tannins and other desirable traits for consumption. With all this knowledge, highly promising projects could be carried out, for example, breeding programs or marker-assisted selection with some elite cultivars.
